# Supplementary material for: Diagnostic performance of deep learning in infectious keratitis: a systematic review and meta-analysis protocol
Source: BMJ Open. 2023 May 10;13(5):e065537. doi: 10.1136/bmjopen-2022-065537 (PMC10173987; doi:10.1136/bmjopen-2022-065537)
Supplement: Author's manuscript [file bmjopen-2022-065537.draft_revisions.pdf]

BMJ Open is committed to open peer review. As part of this commitment we make the peer review history of every article we publish publicly available.

When an article is published we post the peer reviewers' comments and the authors' responses online. We also post the versions of the paper that were used during peer review. These are the versions that the peer review comments apply to.

The versions of the paper that follow are the versions that were submitted during the peer review process. They are not the versions of record or the final published versions. They should not be cited or distributed as the published version of this manuscript.

BMJ Open is an open access journal and the full, final, typeset and author-corrected version of record of the manuscript is available on our site with no access controls, subscription charges or pay-per-view fees (http://bmjopen.bmj.com).

If you have any questions on BMJ Open's open peer review process please email info.bmjopen@bmj.com

BMJ Open

Diagnostic Performance of Deep Learning in Infectious Keratitis: A Systematic Review and Meta-Analysis Protocol

Journal:	*BMJ Open*
Manuscript ID	bmjopen-2022-065537
Article Type:	Protocol
Date Submitted by the Author:	08-Jun-2022
Complete List of Authors:	Ong, Zun Zheng; Queen's Medical Centre<br>Sadek, Youssef; Queen's Medical Centre<br>Liu, Xiaoxuan; University of Birmingham,<br>Qureshi, Riaz; Johns Hopkins University<br>Liu, Su-Hsun; University of Colorado - Anschutz Medical Campus<br>Li, Tianjing; University of Colorado<br>Sounderajah, Viknesh; Imperial College London<br>Ashrafian, Hutan; Imperial College London, Department of Surgery & Cancer;<br>Ting, Daniel; National University of Singapore,<br>Said, Dalia; Queen's Medical Centre<br>Mehta, Jodhbir; Singapore National Eye Centre, Cornea And Ext Disease<br>Burton, Matthew J; London School of Hygiene and Tropical Medicine<br>Dua, Harminder; Queen's Medical Centre<br>Ting, Darren Shu Jeng; University of Nottingham; Queen's Medical Centre
Keywords:	Corneal and external diseases < OPHTHALMOLOGY, MICROBIOLOGY, Diagnostic microbiology < INFECTIOUS DISEASES, OPHTHALMOLOGY

SCHOLARONE™
Manuscripts

Diagnostic Performance of Deep Learning in Infectious Keratitis: A Systematic Review and Meta-Analysis Protocol

Zun Zheng Ong, MBChB,[1] Youssef Sadek,[1] Xiaoxuan Liu, MBChB, PhD,[2,3,4] Riaz Qureshi, PhD,[5] Su-Hsun Liu, PhD,[6] Tianjing Li, PhD,[6] Viknesh Sounderajah, MD,[7] Hutan Ashrafian, MD, PhD,[7] Daniel S. W. Ting, MD, PhD,[8] Dalia G. Said, FRCOphth, MD,[1,9,10] Jodhbir S. Mehta, FRCOphth, PhD,[8] Matthew J. Burton, FRCOphth, PhD,[11,12] Harminder S. Dua, FRCOphth, PhD,[1,9] Darren S. J. Ting, FRCOphth, PhD[1,9]

[1]Department of Ophthalmology, Queen's Medical Centre, Nottingham, UK.

[2]Academic Unit of Ophthalmology, Institute of Inflammation and Ageing, College of Medical and Dental Sciences, University of Birmingham, UK

[3]Department of Ophthalmology, University Hospitals Birmingham NHS Foundation Trust, Birmingham, UK.

[4]Health Data Research UK, London, UK.

[5]Department of Epidemiology, Johns Hopkins University, Baltimore, MD, USA.

[6]Department of Ophthalmology, University of Colorado Anschutz Medical Campus, Aurora, CO, USA.

[7]Institute of Global Health Innovation, Imperial College London, London, UK.

[8]Singapore National Eye Centre, Singapore Eye Research Institute, Singapore.

[9]Academic Ophthalmology, School of Medicine, University of Nottingham, Nottingham, UK.

[10]Research Institute of Ophthalmology, Giza, Egypt.

[11]International Centre for Eye Health, London School of Hygiene and Tropical Medicine, London, UK.

[12]National Institute for Health Research (NIHR) Biomedical Research Centre, Moorfields Eye Hospital NHS Foundation Trust and UCL Institute of Ophthalmology, London, UK.

For peer review only

For peer review only - http://bmjopen.bmj.com/site/about/guidelines.xhtml

Corresponding author: Darren S. J. Ting

Corresponding address: Academic Ophthalmology, School of Medicine, University of Nottingham, Nottingham, NG7 2RD UK.

Email: ting.darren@gmail.com

Tel: +44 (0) 115 951 5151

For peer review only

ABSTRACT

Introduction: Infectious keratitis (IK) represents the 5th leading cause of blindness worldwide. A delay in diagnosis is often a major factor in progression to irreversible visual impairment and/or blindness from IK. The diagnostic challenge is further compounded by low microbiological culture yield, long turnaround time, poorly differentiated clinical features, and polymicrobial infections. In recent years, deep learning (DL), a subfield of artificial intelligence, has rapidly emerged as a promising tool in assisting automated medical diagnosis, clinical triage and decision making, and improving workflow efficiency in healthcare services. Recent studies have demonstrated the potential of using DL in assisting the diagnosis of IK, though the accuracy remains to be elucidated. This systematic review and meta-analysis aims to critically examine and compare the performance of various DL models with clinical experts and/or microbiological results (the current "gold standard") in diagnosing IK, with an aim to inform practice on the clinical applicability and deployment of DL-assisted diagnostic models.

Methods and analysis: This review will consider studies that included application of any DL models to diagnose patients with suspected IK, encompassing bacterial, fungal, protozoal and/or viral origins. We will search various electronic databases, including EMBASE and MEDLINE. There will be no restriction to the language and publication date. Two independent reviewers will assess the titles, abstracts and full-text articles. Extracted data will include details of each primary studies, including title, year of publication, authors, types of DL models used, populations, sample size, decision threshold, and diagnostic performance. We will perform meta-analyses for the included primary studies when there are sufficient similarities in outcome reporting.

Ethics and dissemination: No ethical approval is required for this systematic review. We plan to disseminate our findings via presentation/publication in a peer-reviewed journal.

For peer review only - http://bmjopen.bmj.com/site/about/guidelines.xhtml

Protocol registration: This systematic review protocol will be registered with the PROSPERO after peer review.

STRENGTH AND LIMITATIONS OF THIS STUDY

- This study will serve as the most up-to-date systematic review and meta-analysis specifically evaluating the diagnostic performance of deep learning in infectious keratitis.
- The quality of the study will depend on the quality of the available published literature related to this topic.
- This study will help identify the gaps in the current clinical evidence, which may be related to study design, quality of the research methodologies, setting of reference standard, risk of bias, and outcome reporting.

For peer review only

INTRODUCTION

Due to worldwide population ageing and urbanisation, it is expected that close to 900 million people will suffer from distance vision impairment, of whom 61 million people will be blind by 2050.[1] Infectious keratitis (IK), also commonly known as corneal infection, currently represents the 5th leading cause of blindness worldwide.[2, 3] It can be caused by a wide variety of pathogens such as bacteria, fungi, protozoa, and viruses.[3, 4] Once considered a "silent epidemic" in low- and middle-income countries (LMICs), IK has so far caused ~5 million cases of blindness around the world and is estimated to cause ~2 million monocular blindness each year, placing significant burden on global human health.[3, 5] A recent meta-analysis conducted by Brown et al.[6] estimated that the global incidence of fungal keratitis alone (without accounting for other types of IK) is likely >1 million cases per year, primarily affecting the populations in Africa and Asia. Previous studies have also consistently reported a disproportionately higher incidence of IK in the LMICs (113-799 per 100,000 populations-year) than in high-income countries (HICs; 2.5-40.3 per 100,000 populations-year),[3, 7, 8] which was likely attributable to increased risk of trauma from agricultural and other occupational activities, environmental factors, the use of traditional eye medicine (which may contain pathogens) and the limited access to primary and secondary eye care .[3, 9-11]

Patients affected by IK are often debilitated by severe ocular pain and sight loss, and some are at risk of losing the eye due to intractable infection.[6, 11-14] The outcome of IK is critically dependent on a timely and accurate diagnosis, followed by appropriate medical and/or surgical interventions. In current clinical practice, IK is usually diagnosed on clinical grounds with support from additional tests, including microbiological investigations [e.g. smear microscopy, culture and sensitivity testing, and polymerase chain reaction (PCR)] and/or corneal imaging [e.g. *in vivo* confocal microscopy (IVCM)].[15-17] However, these approaches have multiple challenges, including the need for clinical expertise and equipment, low microbiological culture yield, long turnaround time, poorly differentiated clinical features, and polymicrobial infections.[7, 15, 18, 19] Moreover, access to such microbiological and imaging

For peer review only - http://bmjopen.bmj.com/site/about/guidelines.xhtml

investigations is not available in many ophthalmic units in LMICs, leading to a reliance of empirical treatment. This can lead to a misdiagnosis when based on clinical features alone and the use of incorrect antimicrobial therapy (e.g., fungal keratitis being treated only with anti-bacterial agents). This can result in delays in the initiation of effective treatment, with consequent poorer clinical outcomes and higher risk of ocular complications.

In recent years, the interest of integrating AI into clinical medicine with the hope of improving the quality of healthcare services has been reignited,[20] primarily owing to the advancement in deep learning (DL) techniques, improvement in computing power and increased availability of big data.[21-24] DL, a subfield of AI, has demonstrated promise in assisting automated medical diagnosis, clinical triage and decision making, as well as improving the workflow efficiency in healthcare services in both developed and developing countries.[22-28] Within the realm of ophthalmology, DL research previously focussed mainly on various posterior segment diseases (e.g., age-related macular degeneration, diabetic retinopathy, and glaucoma) and demonstrated comparable, if not better, diagnostic accuracy compared to healthcare professionals.[22, 23, 29-31] Although several recent studies have demonstrated the potential of DL in assisting the diagnosis of IK and distinguishing IK from other ocular diseases,[32-35] the diagnostic accuracy of these DL models remains to be elucidated.

To the best of our knowledge, there is no published systematic review and/or meta-analysis specifically evaluating the diagnostic performance of DL in IK. In view of the current diagnostic challenges of IK and the potential of DL in addressing the highlighted limitations, this systematic review and meta-analysis aims to critically examine and compare the performance of various DL models with clinical experts (the current "gold standard") in diagnosing IK, which can help inform the clinical practice on the potential clinical applicability and deployment of these DL model.

For peer review only

REVIEW QUESTIONS / OBJECTIVES

The proposed systematic review aims to answer the following main questions:

1. What is the diagnostic accuracy of DL models in detecting and differentiating IK from healthy eyes?
2. What is the diagnostic performance of DL models in differentiating IK from other types of corneal or ocular diseases?
3. What is the diagnostic accuracy of DL models in differentiating the types of IK (e.g., bacterial keratitis vs. fungal keratitis)?

For peer review only

METHODS

This protocol was produced based on the Preferred Reporting Items for Systematic Review and Meta-Analysis Protocols (PRISMA-P).[36] This systematic review will be conducted in accordance with the recommendations of the Cochrane Handbook for Systematic Reviews of Diagnostic Test Accuracy. We will write the resulting paper following the Preferred Reporting Items for Systematic Reviews and Meta-analyses of Diagnostic Test Accuracy (PRISMA-DTA)[37] and the CHecklist for critical Appraisal and data extraction for systematic Reviews of prediction Modelling Studies (CHARMS).[38]

Eligibility criteria

This diagnostic accuracy systematic review will consider all relevant clinical studies, including prospective and retrospective comparative cohort studies, case-control studies, and cross-sectional studies, that examined the accuracy of DL in diagnosing any types of IK, encompassing bacterial, fungal, acanthamoeba, and/or viral keratitis. We will exclude case reports and reviews. We will only include studies that employed corneal imaging tests, which may include slit-lamp photography, in vivo confocal microscopy (IVCM), anterior segment optical coherence tomography (AS-OCT), and/or corneal topography/tomography. We will exclude AI studies that contained only data without any imaging or those that focused on image segmentation instead of disease classification. There will be no restriction on patients'

For peer review only - http://bmjopen.bmj.com/site/about/guidelines.xhtml

age, gender, ethnicity, and geographical location. There will be no restriction on the number and proportion of images used for each stage of the DL models, including training, validation and testing stages.

Information sources and search strategy

We will search various bibliographic databases, including EMBASE (OVID), MEDLINE (OVID), the Cochrane Central Register of Controlled Trials (CENTRAL), and trial registries including ISRCTN registry (www.isrctn.com/), US National Institutes of Health Ongoing Trials Register (https://www.clinicaltrials.gov/), and World Health Organization (WHO) International Clinical Trials Registry Platform (ICTRP) for primary research related to DL for diagnosing IK. The search strategy aims to locate both published and unpublished studies. The search will be developed with two concepts built into the search strategy to capture relevant articles: (i) artificial intelligence and (ii) infectious keratitis. There will be no restriction on the study design, date or language for the search. The search strategy, including all identified keywords and index terms, will be adapted to each included information source. The reference list of all eligible studies will be manually screened for additional studies. An example of the search strategy is provided in **Table 1**.

Study selection

Following the search, all identified citations will be loaded into EndNote20 (Clarivate Analytics, PA, USA) and duplicates will be removed. The titles and abstract will be screened by two independent reviewers for assessment against the inclusion criteria of the review, using the Rayyan AI platform (Qatar).[39] The full text of selected citations will then be assessed in detail against the inclusion criteria by two independent reviewers. Reasons for any exclusion of full-text studies will be recorded and reported in the systematic review. Any disagreements between reviewers at each stage of study selection will be resolved through discussions or consultations with a third reviewer. Results of the search will be reported in full in the final systematic review and presented in a Preferred Reporting Items for

For peer review only - http://bmjopen.bmj.com/site/about/guidelines.xhtml

Systematic Reviews and Meta-analyses of Diagnostic Test Accuracy (PRISMA-DTA) flow diagram.[37]

Table 1. A summary of the search strategy using EMBASE for studies related to artificial intelligence in diagnosing infectious keratitis.

#	Query	Results up to 8 May 2022
1	exp artificial intelligence/	60,341
2	artificial intelligence.mp.	46,874
3	exp machine learning/	306,312
4	machine learning.mp.	91,038
5	exp deep learning/	23,936
6	deep learning.mp.	36,705
7	machine intelligence.mp.	242
8	exp support vector machine/	30,564
9	support vector machine.mp.	34,400
10	computer-assisted.mp.	943,528
11	visual data exploration.mp.	18
12	1 or 2 or 3 or 4 or 5 or 6 or 7 or 8 or 9 or 10 or 11	1,266,088
13	exp keratitis/	34,942
14	keratitis.mp.	26,696
15	infectious keratitis.mp.	1,369
16	infective keratitis.mp.	130
17	exp microbial keratitis/	8,393
18	microbial keratitis.mp.	1,606
19	corneal infection.mp.	1,037
20	exp cornea ulcer/	7,787
21	corneal ulcer.mp.	2,586
22	13 or 14 or 15 or 16 or 17 or 18 or 19 or 20 or 21	38,271
23	12 and 22	681
24	limit 23 to human	595

For peer review only

Data collection and data items

Data will be extracted from the included articles by two independent reviewers using a standardised and pilot-tested data extraction tool, RevMan 5.4 (Copenhagen: The Nordic Cochrane Centre, Cochrane). The extracted data will include specific details about the name of authors, study title, year of publication, countries of study, populations (including diseased and healthy cases), demographic factors (i.e. age, gender, ethnicity), sample size, study methods, types of DL algorithms, decision threshold, types of reference standard [which may include expert consensus, microbiological results confirmed on either smear microscopy, culture or polymerase chain reaction (PCR), and/or corneal imaging such as IVCM], and diagnostic accuracy (including the sensitivity and specificity) of the index test (i.e. DL algorithms) and the comparator (i.e. non-expert healthcare professionals), if available. We will extract sufficient information to build 2x2 contingency tables at the reported threshold for each study. The contingency tables will include true positive, false positive, true negative, and false negative to calculate the sensitivity and specificity. If various contingency tables were provided for the same or different algorithms in the same study, they are assumed to be independent from each other as the aim of this work is to provide an overview of the results of various studies instead of the precise point estimates.[28] Any disagreements will be resolved by group consensus. Authors of eligible studies will be contacted to request any missing data, where required.

Outcomes

The primary outcome will be the diagnostic accuracy of DL algorithms in distinguishing IK from healthy eyes and/or those with other types of corneal diseases, as compared to the reference standard. The diagnostic accuracy of each group will be presented in the form of sensitivity and specificity.[40] The secondary outcomes for this review will involve a comparison of the accuracy in differentiating various types of IK and in differentiating IK from other types of corneal or ocular surface diseases. For studies that focused on distinguishing IK (any type) from healthy corneas or those with other non-IK corneal pathologies, the

For peer review only - http://bmjopen.bmj.com/site/about/guidelines.xhtml

reference standard will be the expert consensus and/or microbiological results. For studies that focused on differentiating the subtypes of organisms (e.g., bacteria vs. fungi), the microbiological results or expert consensus (if microscopy or culture results were not available) will be the reference standard. Other potential outcomes for this review will include the accuracy of DL in predicting the culture positivity and clinical outcomes of IK based on the initial presenting images. These secondary outcomes may not be feasible if these more specific questions are not ascertained in the included studies, but they are nonetheless of interest.

Risk of bias assessment

Eligible studies will be critically appraised by two independent reviewers at the outcome level for methodological quality in the review using the Quality Assessment of Diagnostic Accuracy Studies-2 (QUADAS-2) tool.[41] QUADAS-AI (an AI-specific extension to QUADAS-2) tool[42] will be used if available by the time of the conduct of this systematic review. Specifically, we will assess the Risk of Bias for our primary outcomes (i.e., accuracy of DL versus reference standard for IK). The questions used in these tools are split into four domains: patient selection, index test, reference test, and flow and timing. Each of these domain help assess the risk of bias created by patient selection, the conduct and interpretation of index test and reference test and the sequence and timing of the study respectively. We will also assess whether the AI systems have been tested on an externally validated test set. Authors of papers will be contacted to request for additional data for clarification, where necessary. Any disagreements will be resolved by discussion or by seeking the advice of a third reviewer. All studies, regardless of the results of their methodological quality, will undergo data extraction and synthesis. The results of critical appraisal will be reported in the final systematic review, in both narrative and tabular formats.

For peer review only

Data synthesis and analysis

The analysis will be conducted at two levels; (1) a systematic synthesis of all eligible studies; and (2) a meta-analysis of all relevant studies with similar outcome reporting. For the meta-analysis, the intervention group (i.e., the "index test") will refer to the image-based DL algorithms for diagnosing or differentiating IK from other ocular diseases. The reference group will be the expert consensus and/or microbiological results, also known as the gold standard or "ground truth" for the DL algorithms whereas the comparator group, if available, will be the non-expert healthcare professionals.

Where possible, we will pool similar measures of accuracy from studies with statistical meta-analysis using RevMan 5.4 software. 'Paired' forest plots, with one forest plot for sensitivity and the other for specificity, will be used and presented side by side.[43] The means and 95% confidence intervals of each selected primary studies will be presented alongside the number of true positives, false positives, true negatives, and false negatives, wherever appropriate. Summary Receiver Operating Characteristic (SROC) curves will also be plotted using the sensitivity and specificity of each primary study. Chi-square or Fisher exact tests will be used to assess the heterogeneity objectively, if needed.[43] We expect heterogeneity in the types of DL systems and algorithms used across studies and we will consider all to be acceptable "interventions" for analysis as our question is meant to assess the general accuracy of any DL system. In view of the anticipated inter-studies heterogeneity, a random-effects model will be used for the meta-analysis to determine the pooled sensitivity and specificity of the included studies. A fixed-effect model may be used if there is no significant heterogeneity. Subgroup analyses will be conducted where there is sufficient data to investigate different types of IK and other ocular surface disease, as per our pre-specified secondary outcomes.

The heterogeneity between studies will be assessed through the graphic display of paired forest plots or SROC curves. We will evaluate potential publication bias of the pooled data

For peer review only

For peer review only - http://bmjopen.bmj.com/site/about/guidelines.xhtml

using Deek's funnel plot, and p<0.05 will be considered of significant publication bias.[44, 45] The Grading of Recommendations, Assessment, Development and Evaluation (GRADE) approach for grading the certainty of evidence will be followed.[36] A Summary of Findings (SoF) table, created using GRADEpro software, will be presented. Where appropriate, the following information will be included in the SoF: number and type(s) of studies contributing to the outcome, total sample size contributing to the outcome, ranking of the certainty of the evidence based on the risk of bias, heterogeneity, directness, publication bias and precision of the review results. We will include the following outcomes in the SoF table: AUC, sensitivity, and specificity for IK overall (i.e., primary outcome).

Patient and public involvement

DSJT had previously involved patients who were affected by IK to help identify the research need and priority in relation to IK. Many of the patients with IK have highlighted the importance of timely and accurate diagnosis of IK as the delay in diagnosis has negatively affected their visual outcomes. This serves as one of the key reasons for conducting this systematic review and meta-analysis, which aims to improve the diagnosis of IK in clinical settings.

Clinical relevance of this systematic review

The results of this systematic review and meta-analysis will provide high-quality evidence on the diagnostic accuracy of DL in IK. This study will help identify the gaps (if any) in the current clinical evidence, which may be related to study design, quality of the research methodologies, setting of reference standard, risk of bias, and outcome reporting. The identification of these issues can help refine the study design of any future clinical trials evaluating the diagnostic accuracy of DL in IK in a real-world setting. These findings will also help inform the clinicians, researchers, policy makers and regulatory bodies on the clinical applicability of DL in diagnosing IK, with an aim to develop more accessible investigations for IK in the future, including in both HIC and LMICs.

For peer review only - http://bmjopen.bmj.com/site/about/guidelines.xhtml

Financial Support / Funding: MJB is supported by the Wellcome Trust (Grant number 207472/Z/17/Z). DSJT is supported by Medical Research Council / Fight for Sight Clinical Research Fellowship (MR/T001674/1). The funder has not role in developing this protocol.

Author contributions: Study design and conceptualisation: DSJT; Development of study protocol: All authors; Data collection: ZZO, YS; Drafting of initial manuscript: ZZO, DSJT; Review of the manuscript: All authors; Guarantor of the study: DSJT

Conflict of Interest: None to declare.

For peer review only

REFERENCES

1. Burton MJ, Ramke J, Marques AP, Bourne RRA, Congdon N, Jones I, et al. The Lancet Global Health Commission on Global Eye Health: vision beyond 2020. Lancet Glob Health. 2021;9(4):e489-e551.
2. Flaxman SR, Bourne RRA, Resnikoff S, Ackland P, Braithwaite T, Cicinelli MV, et al. Global causes of blindness and distance vision impairment 1990–2020: a systematic review and meta-analysis. The Lancet Global Health. 2017;5(12):e1221-e34.
3. Ting DSJ, Ho CS, Deshmukh R, Said DG, Dua HS. Infectious keratitis: an update on epidemiology, causative microorganisms, risk factors, and antimicrobial resistance. Eye (Lond). 2021;35(4):1084-101.
4. Durand ML, Barshak MB, Chodosh J. Infectious Keratitis in 2021. JAMA. 2021;326(13):1319-20.
5. Ung L, Acharya NR, Agarwal T, Alfonso EC, Bagga B, Bispo PJ, et al. Infectious corneal ulceration: a proposal for neglected tropical disease status. Bull World Health Organ. 2019;97(12):854-6.

6. Brown L, Leck AK, Gichangi M, Burton MJ, Denning DW. The global incidence and diagnosis of fungal keratitis. Lancet Infect Dis. 2021;21(3):e49-e57.
7. Ting DSJ, Ho CS, Cairns J, Elsahn A, Al-Aqaba M, Boswell T, et al. 12-year analysis of incidence, microbiological profiles and in vitro antimicrobial susceptibility of infectious keratitis: the Nottingham Infectious Keratitis Study. Br J Ophthalmol. 2021;105(3):328-33.
8. Collier SA, Gronostaj MP, MacGurn AK, Cope JR, Awsumb KL, Yoder JS, et al. Estimated burden of keratitis--United States, 2010. MMWR Morb Mortal Wkly Rep. 2014;63(45):1027-30.
9. Gopinathan U, Sharma S, Garg P, Rao GN. Review of epidemiological features, microbiological diagnosis and treatment outcome of microbial keratitis: experience of over a decade. Indian J Ophthalmol. 2009;57(4):273-9.
10. Chidambaram JD, Venkatesh Prajna N, Srikanthi P, Lanjewar S, Shah M, Elakkiya S, et al. Epidemiology, risk factors, and clinical outcomes in severe microbial keratitis in South India. Ophthalmic Epidemiol. 2018;25(4):297-305.
11. Ting DSJ, Cairns J, Gopal BP, Ho CS, Krstic L, Elsahn A, et al. Risk Factors, Clinical Outcomes, and Prognostic Factors of Bacterial Keratitis: The Nottingham Infectious Keratitis Study. Front Med (Lausanne). 2021;8:715118.
12. Ting DSJ, Galal M, Kulkarni B, Elalfy MS, Lake D, Hamada S, et al. Clinical Characteristics and Outcomes of Fungal Keratitis in the United Kingdom 2011-2020: A 10-Year Study. J Fungi (Basel). 2021;7(11).
13. Khor WB, Prajna VN, Garg P, Mehta JS, Xie L, Liu Z, et al. The Asia Cornea Society Infectious Keratitis Study: A Prospective Multicenter Study of Infectious Keratitis in Asia. Am J Ophthalmol. 2018;195:161-70.
14. Ting DSJ, McKenna M, Sadiq SN, Martin J, Mudhar HS, Meeney A, et al. Arthrographis kalrae Keratitis Complicated by Endophthalmitis: A Case Report With Literature Review. Eye Contact Lens. 2020;46(6):e59-e65.

For peer review only

15. Ting DSJ, Gopal BP, Deshmukh R, Seitzman GD, Said DG, Dua HS. Diagnostic armamentarium of infectious keratitis: A comprehensive review. Ocul Surf. 2022;23:27-39.
16. Bakken IM, Jackson CJ, Utheim TP, Villani E, Hamrah P, Kheirkhah A, et al. The use of in vivo confocal microscopy in fungal keratitis - Progress and challenges. Ocul Surf. 2022;24:103-18.
17. Ting DSJ, Said DG, Dua HS. Interface Haze After Descemet Stripping Automated Endothelial Keratoplasty. JAMA Ophthalmol. 2019;137(10):1201-2.
18. Khoo P, Cabrera-Aguas MP, Nguyen V, Lahra MM, Watson SL. Microbial keratitis in Sydney, Australia: risk factors, patient outcomes, and seasonal variation. Graefes Arch Clin Exp Ophthalmol. 2020;258(8):1745-55.
19. Ting DSJ, Bignardi G, Koerner R, Irion LD, Johnson E, Morgan SJ, et al. Polymicrobial Keratitis With Cryptococcus curvatus, Candida parapsilosis, and Stenotrophomonas maltophilia After Penetrating Keratoplasty: A Rare Case Report With Literature Review. Eye Contact Lens. 2019;45(2):e5-e10.
20. Schwalbe N, Wahl B. Artificial intelligence and the future of global health. Lancet. 2020;395(10236):1579-86.
21. LeCun Y, Bengio Y, Hinton G. Deep learning. Nature. 2015;521(7553):436-44.
22. Ting DSW, Cheung CY-L, Lim G, Tan GSW, Quang ND, Gan A, et al. Development and Validation of a Deep Learning System for Diabetic Retinopathy and Related Eye Diseases Using Retinal Images From Multiethnic Populations With Diabetes. JAMA. 2017;318(22):2211-23.
23. De Fauw J, Ledsam JR, Romera-Paredes B, Nikolov S, Tomasev N, Blackwell S, et al. Clinically applicable deep learning for diagnosis and referral in retinal disease. Nat Med. 2018;24(9):1342-50.
24. Seah JCY, Tang CHM, Buchlak QD, Holt XG, Wardman JB, Aimoldin A, et al. Effect of a comprehensive deep-learning model on the accuracy of chest x-ray interpretation by

For peer review only

radiologists: a retrospective, multireader multicase study. Lancet Digit Health. 2021;3(8):e496-e506.

25. Soenksen LR, Kassis T, Conover ST, Marti-Fuster B, Birkenfeld JS, Tucker-Schwartz J, et al. Using deep learning for dermatologist-level detection of suspicious pigmented skin lesions from wide-field images. Sci Transl Med. 2021;13(581).
26. Sabanayagam C, Xu D, Ting DSW, Nusinovici S, Banu R, Hamzah H, et al. A deep learning algorithm to detect chronic kidney disease from retinal photographs in community-based populations. Lancet Digit Health. 2020;2(6):e295-e302.
27. Ting DSJ, Ang M, Mehta JS, Ting DSW. Artificial intelligence-assisted telemedicine platform for cataract screening and management: a potential model of care for global eye health. Br J Ophthalmol. 2019;103(11):1537-8.
28. Liu X, Faes L, Kale AU, Wagner SK, Fu DJ, Bruynseels A, et al. A comparison of deep learning performance against health-care professionals in detecting diseases from medical imaging: a systematic review and meta-analysis. Lancet Digit Health. 2019;1(6):e271-e97.
29. Gulshan V, Peng L, Coram M, Stumpe MC, Wu D, Narayanaswamy A, et al. Development and Validation of a Deep Learning Algorithm for Detection of Diabetic Retinopathy in Retinal Fundus Photographs. JAMA. 2016;316(22):2402-10.
30. Taylor S, Brown JM, Gupta K, Campbell JP, Ostmo S, Chan RVP, et al. Monitoring Disease Progression With a Quantitative Severity Scale for Retinopathy of Prematurity Using Deep Learning. JAMA Ophthalmology. 2019;137(9):1022-8.
31. Li JO, Liu H, Ting DSJ, Jeon S, Chan RVP, Kim JE, et al. Digital technology, tele-medicine and artificial intelligence in ophthalmology: A global perspective. Prog Retin Eye Res. 2021;82:100900.
32. Li W, Yang Y, Zhang K, Long E, He L, Zhang L, et al. Dense anatomical annotation of slit-lamp images improves the performance of deep learning for the diagnosis of ophthalmic disorders. Nat Biomed Eng. 2020;4(8):767-77.

For peer review only

33. Tiwari M, Piech C, Baitemirova M, Prajna NV, Srinivasan M, Lalitha P, et al. Differentiation of Active Corneal Infections from Healed Scars Using Deep Learning. Ophthalmology. 2022;129(2):139-46.

34. Koyama A, Miyazaki D, Nakagawa Y, Ayatsuka Y, Miyake H, Ehara F, et al. Determination of probability of causative pathogen in infectious keratitis using deep learning algorithm of slit-lamp images. Sci Rep. 2021;11(1):22642.

35. Ting DSJ, Foo VH, Yang LWY, Sia JT, Ang M, Lin H, et al. Artificial intelligence for anterior segment diseases: Emerging applications in ophthalmology. Br J Ophthalmol. 2021;105(2):158-68.

36. Shamseer L, Moher D, Clarke M, Ghersi D, Liberati A, Petticrew M, et al. Preferred reporting items for systematic review and meta-analysis protocols (PRISMA-P) 2015: elaboration and explanation. BMJ. 2015;349:g7647.

37. Salameh J-P, Bossuyt PM, McGrath TA, Thombs BD, Hyde CJ, Macaskill P, et al. Preferred reporting items for systematic review and meta-analysis of diagnostic test accuracy studies (PRISMA-DTA): explanation, elaboration, and checklist. BMJ. 2020;370:m2632.

38. Moons KG, de Groot JA, Bouwmeester W, Vergouwe Y, Mallett S, Altman DG, et al. Critical appraisal and data extraction for systematic reviews of prediction modelling studies: the CHARMS checklist. PLoS Med. 2014;11(10):e1001744.

39. Ouzzani M, Hammady H, Fedorowicz Z, Elmagarmid A. Rayyan-a web and mobile app for systematic reviews. Syst Rev. 2016;5(1):210.

40. Rampat R, Deshmukh R, Chen X, Ting DSW, Said DG, Dua HS, et al. Artificial Intelligence in Cornea, Refractive Surgery, and Cataract: Basic Principles, Clinical Applications, and Future Directions. Asia Pac J Ophthalmol (Phila). 2021;10(3):268-81.

41. Whiting PF, Rutjes AW, Westwood ME, Mallett S, Deeks JJ, Reitsma JB, et al. QUADAS-2: a revised tool for the quality assessment of diagnostic accuracy studies. Ann Intern Med. 2011;155(8):529-36.

For peer review only

42. Sounderajah V, Ashrafian H, Rose S, Shah NH, Ghassemi M, Golub R, et al. A quality assessment tool for artificial intelligence-centered diagnostic test accuracy studies: QUADAS-AI. Nat Med. 2021;27(10):1663-5.

43. Campbell JM, Klugar M, Ding S, Carmody DP, Hakonsen SJ, Jadotte YT, et al. Diagnostic test accuracy: methods for systematic review and meta-analysis. JBI Evidence Implementation. 2015;13(3).

44. Deeks JJ, Macaskill P, Irwig L. The performance of tests of publication bias and other sample size effects in systematic reviews of diagnostic test accuracy was assessed. J Clin Epidemiol. 2005;58(9):882-93.

45. van Enst WA, Ochodo E, Scholten RJ, Hooft L, Leeflang MM. Investigation of publication bias in meta-analyses of diagnostic test accuracy: a meta-epidemiological study. BMC Med Res Methodol. 2014;14:70.

For peer review only

PRISMA-P (Preferred Reporting Items for Systematic review and Meta-Analysis Protocols) 2015 checklist: recommended items to address in a systematic review protocol*

Section and topic	Item No	Checklist item	Page
**ADMINISTRATIVE INFORMATION**			
Title:			
Identification	1a	Identify the report as a protocol of a systematic review	1
Update	1b	If the protocol is for an update of a previous systematic review, identify as such	N/A
Registration	2	If registered, provide the name of the registry (such as PROSPERO) and registration number	N/A
Authors:			
Contact	3a	Provide name, institutional affiliation, e-mail address of all protocol authors; provide physical mailing address of corresponding author	2
Contributions	3b	Describe contributions of protocol authors and identify the guarantor of the review	12
Amendments	4	If the protocol represents an amendment of a previously completed or published protocol, identify as such and list changes; otherwise, state plan for documenting important protocol amendments	N/A
Support:			
Sources	5a	Indicate sources of financial or other support for the review	2
Sponsor	5b	Provide name for the review funder and/or sponsor	2
Role of sponsor or funder	5c	Describe roles of funder(s), sponsor(s), and/or institution(s), if any, in developing the protocol	2
**INTRODUCTION**			
Rationale	6	Describe the rationale for the review in the context of what is already known	5-6
Objectives	7	Provide an explicit statement of the question(s) the review will address with reference to participants, interventions, comparators, and outcomes (PICO)	7
**METHODS**			
Eligibility criteria	8	Specify the study characteristics (such as PICO, study design, setting, time frame) and report characteristics (such as years considered, language, publication status) to be used as criteria for eligibility for the review	7-8
Information sources	9	Describe all intended information sources (such as electronic databases, contact with study authors, trial registers or other grey literature sources) with planned dates of coverage	8
Search strategy	10	Present draft of search strategy to be used for at least one electronic database, including planned limits, such that it could be repeated	8

For peer review only

Study records:			
Data management	11a	Describe the mechanism(s) that will be used to manage records and data throughout the review	9
Selection process	11b	State the process that will be used for selecting studies (such as two independent reviewers) through each phase of the review (that is, screening, eligibility and inclusion in meta-analysis)	8-9
Data collection process	11c	Describe planned method of extracting data from reports (such as piloting forms, done independently, in duplicate), any processes for obtaining and confirming data from investigators	9
Data items	12	List and define all variables for which data will be sought (such as PICO items, funding sources), any pre-planned data assumptions and simplifications	9
Outcomes and prioritization	13	List and define all outcomes for which data will be sought, including prioritization of main and additional outcomes, with rationale	9-10
Risk of bias in individual studies	14	Describe anticipated methods for assessing risk of bias of individual studies, including whether this will be done at the outcome or study level, or both; state how this information will be used in data synthesis	10
Data synthesis	15a	Describe criteria under which study data will be quantitatively synthesised	11
	15b	If data are appropriate for quantitative synthesis, describe planned summary measures, methods of handling data and methods of combining data from studies, including any planned exploration of consistency (such as I^2, Kendall's τ)	11
	15c	Describe any proposed additional analyses (such as sensitivity or subgroup analyses, meta-regression)	11-12
	15d	If quantitative synthesis is not appropriate, describe the type of summary planned	11-12
Meta-bias(es)	16	Specify any planned assessment of meta-bias(es) (such as publication bias across studies, selective reporting within studies)	12
Confidence in cumulative evidence	17	Describe how the strength of the body of evidence will be assessed (such as GRADE)	12

*** It is strongly recommended that this checklist be read in conjunction with the PRISMA-P Explanation and Elaboration (cite when available) for important clarification on the items. Amendments to a review protocol should be tracked and dated. The copyright for PRISMA-P (including checklist) is held by the PRISMA-P Group and is distributed under a Creative Commons Attribution Licence 4.0.**

From: Shamseer L, Moher D, Clarke M, Ghersi D, Liberati A, Petticrew M, Shekelle P, Stewart L, PRISMA-P Group. Preferred reporting items for systematic review and meta-analysis protocols (PRISMA-P) 2015: elaboration and explanation. BMJ. 2015 Jan 2;349(jan02 1):g7647.

For peer review only

BMJ Open

Diagnostic Performance of Deep Learning in Infectious Keratitis: A Systematic Review and Meta-Analysis Protocol

Journal:	*BMJ Open*
Manuscript ID	bmjopen-2022-065537.R1
Article Type:	Protocol
Date Submitted by the Author:	26-Feb-2023
Complete List of Authors:	Ong, Zun Zheng; Queen's Medical Centre<br>Sadek, Youssef; Queen's Medical Centre<br>Liu, Xiaoxuan; University of Birmingham,<br>Qureshi, Riaz; Johns Hopkins University<br>Liu, Su-Hsun; University of Colorado - Anschutz Medical Campus<br>Li, Tianjing; University of Colorado<br>Sounderajah, Viknesh; Imperial College London<br>Ashrafian, Hutan; Imperial College London, Department of Surgery & Cancer;<br>Ting, Daniel; National University of Singapore,<br>Said, Dalia; Queen's Medical Centre<br>Mehta, Jodhbir; Singapore National Eye Centre, Cornea And Ext Disease<br>Burton, Matthew J; London School of Hygiene and Tropical Medicine<br>Dua, Harminder; Queen's Medical Centre<br>Ting, Darren Shu Jeng; University of Nottingham; University of Birmingham
**Primary Subject Heading**:	Ophthalmology
Secondary Subject Heading:	Diagnostics, Infectious diseases, Evidence based practice
Keywords:	Corneal and external diseases < OPHTHALMOLOGY, MICROBIOLOGY, Diagnostic microbiology < INFECTIOUS DISEASES, OPHTHALMOLOGY, Diagnostic Imaging

SCHOLARONE™
Manuscripts

Diagnostic Performance of Deep Learning in Infectious Keratitis: A Systematic Review and Meta-Analysis Protocol

Zun Zheng Ong, MBChB,[1] Youssef Sadek,[1] Xiaoxuan Liu, MBChB, PhD,[2,3,4] Riaz Qureshi, PhD,[5] Su-Hsun Liu, PhD,[6] Tianjing Li, PhD,[6] Viknesh Sounderajah, MD,[7] Hutan Ashrafian, MD, PhD,[7] Daniel S. W. Ting, MD, PhD,[8] Dalia G. Said, FRCOphth, MD,[1,9,10] Jodhbir S. Mehta, FRCOphth, PhD,[8] Matthew J. Burton, FRCOphth, PhD,[11,12] Harminder S. Dua, FRCOphth, PhD,[1,9] Darren S. J. Ting, FRCOphth, PhD[2,9,13]

[1]Department of Ophthalmology, Queen's Medical Centre, Nottingham, UK.

[2]Academic Unit of Ophthalmology, Institute of Inflammation and Ageing, College of Medical and Dental Sciences, University of Birmingham, UK

[3]Department of Ophthalmology, University Hospitals Birmingham NHS Foundation Trust, Birmingham, UK.

[4]Health Data Research UK, London, UK.

[5]Department of Epidemiology, Johns Hopkins University, Baltimore, MD, USA.

[6]Department of Ophthalmology, University of Colorado Anschutz Medical Campus, Aurora, CO, USA.

[7]Institute of Global Health Innovation, Imperial College London, London, UK.

[8]Singapore National Eye Centre, Singapore Eye Research Institute, Singapore.

[9]Academic Ophthalmology, School of Medicine, University of Nottingham, Nottingham, UK.

[10]Research Institute of Ophthalmology, Giza, Egypt.

[11]International Centre for Eye Health, London School of Hygiene and Tropical Medicine, London, UK.

[12]National Institute for Health Research (NIHR) Biomedical Research Centre, Moorfields Eye Hospital NHS Foundation Trust and UCL Institute of Ophthalmology, London, UK.

[13]Birmingham and Midland Eye Centre, Birmingham, UK.

For peer review only

For peer review only - http://bmjopen.bmj.com/site/about/guidelines.xhtml

Corresponding author: Darren S. J. Ting

Corresponding address: Academic Unit of Ophthalmology, Institute of Inflammation and Ageing, College of Medical and Dental Sciences, University of Birmingham, UK.

Email: ting.darren@gmail.com

Tel: +44 (0) 121 414 3344

For peer review only

ABSTRACT

Introduction: Infectious keratitis (IK) represents the 5th leading cause of blindness worldwide. A delay in diagnosis is often a major factor in progression to irreversible visual impairment and/or blindness from IK. The diagnostic challenge is further compounded by low microbiological culture yield, long turnaround time, poorly differentiated clinical features, and polymicrobial infections. In recent years, deep learning (DL), a subfield of artificial intelligence, has rapidly emerged as a promising tool in assisting automated medical diagnosis, clinical triage and decision making, and improving workflow efficiency in healthcare services. Recent studies have demonstrated the potential of using DL in assisting the diagnosis of IK, though the accuracy remains to be elucidated. This systematic review and meta-analysis aims to critically examine and compare the performance of various DL models with clinical experts and/or microbiological results (the current "gold standard") in diagnosing IK, with an aim to inform practice on the clinical applicability and deployment of DL-assisted diagnostic models.

Methods and analysis: This review will consider studies that included application of any DL models to diagnose patients with suspected IK, encompassing bacterial, fungal, protozoal and/or viral origins. We will search various electronic databases, including EMBASE and MEDLINE, and trial registries. There will be no restriction to the language and publication date. Two independent reviewers will assess the titles, abstracts and full-text articles. Extracted data will include details of each primary studies, including title, year of publication, authors, types of DL models used, populations, sample size, decision threshold, and diagnostic performance. We will perform meta-analyses for the included primary studies when there are sufficient similarities in outcome reporting.

Ethics and dissemination: No ethical approval is required for this systematic review. We plan to disseminate our findings via presentation/publication in a peer-reviewed journal.

For peer review only

Protocol registration: This systematic review has been registered with the PROSPERO (CRD42022348596).

STRENGTH AND LIMITATIONS OF THIS STUDY

- This systematic review and meta-analysis will include all relevant articles related to the diagnostic performance of deep learning in infectious keratitis through a comprehensive search of all literature.
- This review will examine the diagnostic performance of deep learning in identifying and differentiating the subtypes of infectious keratitis, including bacterial, fungal, viral, and parasitic keratitis.
- This review will examine the diagnostic accuracy of deep learning in distinguishing infectious keratitis from healthy eyes and other ocular surface diseases.
- Meta-analysis will be performed to determine the pooled diagnostic sensitivity and specificity of deep learning and will be compared with the performance of healthcare professionals (if data are available).
- The quality of the study will depend on the quality of the available published literature related to this topic.

For peer review only

INTRODUCTION

Due to worldwide population ageing and urbanisation, it is expected that close to 900 million people will suffer from distance vision impairment, of whom 61 million people will be blind by 2050.[1] Infectious keratitis (IK), also commonly known as corneal infection, currently represents the 5th leading cause of blindness worldwide.[2,3] It can be caused by a wide variety of pathogens such as bacteria, fungi, protozoa, and viruses.[3,4] Once considered a "silent epidemic" in low- and middle-income countries (LMICs), IK has so far caused ~5 million cases of blindness around the world and is estimated to cause ~2 million monocular blindness each year, placing significant burden on global human health.[3,5] A recent meta-analysis conducted by Brown et al.[6] estimated that the global incidence of fungal keratitis alone (without accounting for other types of IK) is likely >1 million cases per year, primarily affecting the populations in Africa and Asia. Previous studies have also consistently reported a disproportionately higher incidence of IK in the LMICs (113-799 per 100,000 populations-year) than in high-income countries (HICs; 2.5-40.3 per 100,000 populations-year),[3,7,8] which was likely attributable to increased risk of trauma from agricultural and other occupational activities, environmental factors, the use of traditional eye medicine (which may contain pathogens) and the limited access to primary and secondary eye care.[3,9-11]

Patients affected by IK are often debilitated by severe ocular pain and sight loss, and some are at risk of losing the eye due to intractable infection.[6,11-14] The outcome of IK is critically dependent on a timely and accurate diagnosis, followed by appropriate medical and/or surgical interventions. In current clinical practice, IK is usually diagnosed on clinical grounds with support from additional tests, including microbiological investigations [e.g. smear microscopy, culture and sensitivity testing, and polymerase chain reaction (PCR)] and/or corneal imaging [e.g. *in vivo* confocal microscopy (IVCM)].[15-17] However, these approaches have multiple challenges, including the need for clinical expertise and equipment, low microbiological culture yield, long turnaround time, poorly differentiated clinical features, and polymicrobial infections.[7,15,18,19] Moreover, access to such

For peer review only - http://bmjopen.bmj.com/site/about/guidelines.xhtml

microbiological and imaging investigations is not available in many ophthalmic units in LMICs, leading to a reliance of empirical treatment. This can lead to a misdiagnosis when based on clinical features alone and the use of incorrect antimicrobial therapy (e.g., fungal keratitis being treated only with anti-bacterial agents). This can result in delays in the initiation of effective treatment, with consequent poorer clinical outcomes and higher risk of ocular complications.

In recent years, the interest of integrating AI into clinical medicine with the hope of improving the quality of healthcare services has been reignited,[20] primarily owing to the advancement in deep learning (DL) techniques, improvement in computing power and increased availability of big data.[21-24] DL, a subfield of AI, has demonstrated promise in assisting automated medical diagnosis, clinical triage and decision making, as well as improving the workflow efficiency in healthcare services in both developed and developing countries.[22-28] Within the realm of ophthalmology, DL research previously focussed mainly on various posterior segment diseases (e.g., age-related macular degeneration, diabetic retinopathy, and glaucoma) and demonstrated comparable, if not better, diagnostic accuracy compared to healthcare professionals.[22,23,29-31] Although several recent studies have demonstrated the potential of DL in assisting the diagnosis of IK and distinguishing IK from other ocular diseases,[32-37] the diagnostic accuracy of these DL models remains to be elucidated.

To the best of our knowledge, there is no published systematic review and/or meta-analysis specifically evaluating the diagnostic performance of DL in IK. In view of the current diagnostic challenges of IK and the potential of DL in addressing the highlighted limitations, this systematic review and meta-analysis aims to critically examine and compare the performance of various DL models with clinical experts (the current “gold standard”) in diagnosing IK, which can help inform the clinical practice on the potential clinical applicability and deployment of these DL model.

For peer review only - http://bmjopen.bmj.com/site/about/guidelines.xhtml

REVIEW QUESTIONS / OBJECTIVES

The proposed systematic review aims to answer the following main questions:

1. What is the diagnostic accuracy of DL models in detecting and differentiating IK from healthy eyes?
2. What is the diagnostic performance of DL models in differentiating IK from other types of corneal or ocular diseases?
3. What is the diagnostic accuracy of DL models in differentiating the types of IK (e.g., bacterial keratitis vs. fungal keratitis)?

METHODS

This protocol was produced based on the Preferred Reporting Items for Systematic Review and Meta-Analysis Protocols (PRISMA-P).[38] This systematic review will be conducted in accordance with the recommendations of the Cochrane Handbook for Systematic Reviews of Diagnostic Test Accuracy. We will write the resulting paper following the Preferred Reporting Items for Systematic Reviews and Meta-analyses of Diagnostic Test Accuracy (PRISMA-DTA) [39] and the CHecklist for critical Appraisal and data extraction for systematic Reviews of prediction Modelling Studies (CHARMS).[40]

Eligibility criteria

This diagnostic accuracy systematic review will consider all relevant clinical studies, including prospective and retrospective comparative cohort studies, case-control studies, and cross-sectional studies, that examined the accuracy of DL in diagnosing any types of IK, encompassing bacterial, fungal, acanthamoeba, and/or viral keratitis. We will exclude case reports and reviews. We will only include studies that employed corneal imaging tests, which may include slit-lamp photography, in vivo confocal microscopy (IVCM), anterior segment optical coherence tomography (AS-OCT), and/or corneal topography/tomography. We will exclude AI studies that contained only data without any imaging or those that focused on image segmentation instead of disease classification. There will be no restriction on patients'

For peer review only

age, gender, ethnicity, and geographical location. There will be no restriction on the number and proportion of images used for each stage of the DL models, including training, validation and testing stages.

Information sources and search strategy

We will search various bibliographic databases, including EMBASE (OVID), MEDLINE (OVID), CINAHL, and DANS EASY Archive, and trial registries, including the Cochrane Central Register of Controlled Trials (CENTRAL), ISRCTN registry (www.isrctn.com/), US National Institutes of Health Ongoing Trials Register (https://www.clinicaltrials.gov/), and World Health Organization (WHO) International Clinical Trials Registry Platform (ICTRP) for primary research related to DL for diagnosing IK. The search strategy aims to locate both published and unpublished studies. The search will be developed with two concepts built into the search strategy to capture relevant articles: (i) artificial intelligence and (ii) infectious keratitis. There will be no restriction on the study design, date or language for the search. The search strategy, including all identified keywords and index terms, will be adapted to each included information source. The reference list of all eligible studies will be manually screened for additional studies. An example of the search strategy is provided in **Table 1**.

Study selection

Following the search, all identified citations will be loaded into EndNote X9 (Clarivate Analytics, PA, USA) and duplicates will be removed. The titles and abstract will be screened by two independent reviewers for assessment against the inclusion criteria of the review, using the Rayyan AI platform (Qatar).[41] The full text of selected citations will then be assessed in detail against the inclusion criteria by two independent reviewers. Reasons for exclusion of any full-text studies will be recorded and reported in the systematic review. Any disagreements between reviewers at each stage of study selection will be resolved through discussions or consultations with a third reviewer. The results of the search will be reported in full in the final systematic review and presented in a Preferred Reporting Items for

For peer review only - http://bmjopen.bmj.com/site/about/guidelines.xhtml

Systematic Reviews and Meta-analyses of Diagnostic Test Accuracy (PRISMA-DTA) flow diagram.[39]

Table 1. A summary of the search strategy using EMBASE for studies related to artificial intelligence in diagnosing infectious keratitis.

#	Query	Results from 8 May 2022
1	exp artificial intelligence/	60,341
2	artificial intelligence.mp.	46,874
3	exp machine learning/	306,312
4	machine learning.mp.	91,038
5	exp deep learning/	23,936
6	deep learning.mp.	36,705
7	machine intelligence.mp.	242
8	exp support vector machine/	30,564
9	support vector machine.mp.	34,400
10	computer-assisted.mp.	943,528
11	visual data exploration.mp.	18
12	1 or 2 or 3 or 4 or 5 or 6 or 7 or 8 or 9 or 10 or 11	1,266,088
13	exp keratitis/	34,942
14	keratitis.mp.	26,696
15	infectious keratitis.mp.	1,369
16	infective keratitis.mp.	130
17	exp microbial keratitis/	8,393
18	microbial keratitis.mp.	1,606
19	corneal infection.mp.	1,037
20	exp cornea ulcer/	7,787
21	corneal ulcer.mp.	2,586
22	13 or 14 or 15 or 16 or 17 or 18 or 19 or 20 or 21	38,271
23	12 and 22	681
24	limit 23 to human	595

For peer review only

Data collection and data items

Data will be extracted from the included articles by two independent reviewers using a standardised and pilot-tested data extraction tool, RevMan 5.4 (Copenhagen: The Nordic Cochrane Centre, Cochrane). The extracted data will include specific details about the name of authors, study title, year of publication, countries of study, populations (including diseased and healthy cases), demographic factors (i.e. age, gender, ethnicity), sample size, study methods, types of DL algorithms, decision threshold, types of reference standard [which may include expert consensus, microbiological results confirmed on either smear microscopy, culture or polymerase chain reaction (PCR), and/or corneal imaging such as IVCM], and diagnostic accuracy (including the sensitivity and specificity) of the index test (i.e. DL algorithms) and the comparator (i.e. non-expert healthcare professionals), if available. We will extract sufficient information to build 2x2 contingency tables at the reported threshold for each study. The contingency tables will include true positive, false positive, true negative, and false negative to calculate the sensitivity and specificity. If various contingency tables were provided for the same or different algorithms in the same study, they are assumed to be independent from each other as the aim of this work is to provide an overview of the results of various studies instead of the precise point estimates.[28] Any disagreements will be resolved by group consensus. Authors of eligible studies will be contacted to request any missing data, where required.

Outcomes

The primary outcome will be the diagnostic accuracy of DL algorithms in distinguishing IK from healthy eyes and/or those with other types of corneal diseases, as compared to the reference standard. The diagnostic accuracy of each group will be presented in the form of sensitivity and specificity.[42] The secondary outcomes for this review will involve a comparison of the accuracy in differentiating various types of IK and in differentiating IK from other types of corneal or ocular surface diseases. For studies that focused on distinguishing IK (any type) from healthy corneas or those with other non-IK corneal pathologies, the

For peer review only

reference standard will be the expert consensus and/or microbiological results. For studies that focused on differentiating the subtypes of organisms (e.g., bacteria vs. fungi), the microbiological results or expert consensus (if microscopy or culture results were not available) will be the reference standard. Other potential outcomes for this review will include the accuracy of DL in predicting the culture positivity and clinical outcomes of IK based on the initial presenting images. These secondary outcomes may not be feasible if these more specific questions are not ascertained in the included studies, but they are nonetheless of interest.

Risk of bias assessment

Eligible studies will be critically appraised by two independent reviewers at the outcome level for methodological quality in the review using the Quality Assessment of Diagnostic Accuracy Studies-2 (QUADAS-2) tool.[43] QUADAS-AI (an AI-specific extension to QUADAS-2) tool [44] will be used if available by the time of the conduct of this systematic review. Specifically, we will assess the Risk of Bias for our primary outcomes (i.e., accuracy of DL versus reference standard for IK). The questions used in these tools are split into four domains: patient selection, index test, reference test, and flow and timing. Each of these domain help assess the risk of bias created by patient selection, the conduct and interpretation of index test and reference test and the sequence and timing of the study respectively. We will also assess whether the AI systems have been tested on an externally validated test set. Authors of papers will be contacted to request for additional data for clarification, where necessary. Any disagreements will be resolved by discussion or by seeking the advice of a third reviewer. All studies, regardless of the results of their methodological quality, will undergo data extraction and synthesis. The results of critical appraisal will be reported in the final systematic review, in both narrative and tabular formats.

For peer review only

For peer review only - http://bmjopen.bmj.com/site/about/guidelines.xhtml

Data synthesis and analysis

The analysis will be conducted at two levels; (1) a systematic synthesis of all eligible studies; and (2) a meta-analysis of all relevant studies with similar outcome reporting. For the meta-analysis, the intervention group (i.e., the "index test") will refer to the image-based DL algorithms for diagnosing or differentiating IK from other ocular diseases. The reference group will be the expert consensus and/or microbiological results, also known as the gold standard or "ground truth" for the DL algorithms whereas the comparator group, if available, will be the non-expert healthcare professionals.

Where possible, we will pool similar measures of accuracy from studies with statistical meta-analysis using RevMan 5.4 software. 'Paired' forest plots, with one forest plot for sensitivity and the other for specificity, will be used and presented side by side.[45] The means and 95% confidence intervals of each selected primary studies will be presented alongside the number of true positives, false positives, true negatives, and false negatives, wherever appropriate. Summary Receiver Operating Characteristic (SROC) curves will also be plotted using the sensitivity and specificity of each primary study. Chi-square or Fisher exact tests will be used to assess the heterogeneity objectively, if needed.[45] We expect heterogeneity in the types of DL systems and algorithms used across studies and we will consider all to be acceptable "interventions" for analysis as our question is meant to assess the general accuracy of any DL system. In view of the anticipated inter-studies heterogeneity, a random-effects model will be used for the meta-analysis to determine the pooled sensitivity and specificity of the included studies. A fixed-effect model may be used if there is no significant heterogeneity. Subgroup analyses will be conducted where there is sufficient data to investigate different types of IK and other ocular surface diseases, as per our pre-specified secondary outcomes. Additional subgroup analysis will also be performed on the diagnostic performance of the DL systems based on different imaging modalities, including slit-lamp photography, in vivo confocal microscopy, anterior segment optical coherence tomography, and others.

For peer review only

The heterogeneity between studies will be assessed through the graphic display of paired forest plots or SROC curves. We will evaluate potential publication bias of the pooled data using Deek's funnel plot, and $p<0.05$ will be considered of significant publication bias.[46,47] The Grading of Recommendations, Assessment, Development and Evaluation (GRADE) approach for grading the certainty of evidence will be followed.[38] A Summary of Findings (SoF) table, created using GRADEpro software, will be presented. Where appropriate, the following information will be included in the SoF: number and type(s) of studies contributing to the outcome, total sample size contributing to the outcome, ranking of the certainty of the evidence based on the risk of bias, heterogeneity, directness, publication bias and precision of the review results. We will include the following outcomes in the SoF table: AUC, sensitivity, and specificity for IK overall (i.e., primary outcome).

Patient and public involvement

DSJT had previously involved patients who were affected by IK to help identify the research need and priority in relation to IK. Many of the patients with IK have highlighted the importance of timely and accurate diagnosis of IK as the delay in diagnosis has negatively affected their visual outcomes. This serves as one of the key reasons for conducting this systematic review and meta-analysis, which aims to improve the diagnosis of IK in clinical settings.

Clinical relevance of this systematic review

The results of this systematic review and meta-analysis will provide high-quality evidence on the diagnostic accuracy of DL in IK. This study will help identify the gaps (if any) in the current clinical evidence, which may be related to study design, quality of the research methodologies, setting of reference standard, risk of bias, and outcome reporting. The identification of these issues can help refine the study design of any future clinical trials evaluating the diagnostic accuracy of DL in IK in a real-world setting. These findings will also

For peer review only

help inform the clinicians, researchers, policy makers, and regulatory bodies on the clinical applicability of DL in diagnosing IK, with an aim to develop more accessible investigations for IK in the future, including in both HIC and LMICs.

Financial Support / Funding: MJB is supported by the Wellcome Trust (Grant number 207472/Z/17/Z). DSJT is supported by Medical Research Council / Fight for Sight Clinical Research Fellowship (MR/T001674/1). The funder has not role in developing this protocol.

Authors' contributions: DSJT designed and conceptualized the study. ZZO, YS, XL, RQ, SHL, TL, VS, HA, DSWT, DGS, JSM, MJB, HSD, and DSJT developed the study protocol. ZZO and YS collected the data. ZZO, YS, XL, RQ, SHL, TL, VS, HA, DSWT, DGS, JSM, MJB, HSD, and DSJT interpreted the data. ZZO and DSJT drafted the initial manuscript. ZZO, YS, XL, RQ, SHL, TL, VS, HA, DSWT, DGS, JSM, MJB, HSD, and DSJT critically reviewed, revised and approved the final manuscript. DSJT acts as the guarantor of the study.

Conflict of Interest: None to declare.

For peer review only

References

1. Burton MJ, Ramke J, Marques AP, Bourne RRA, Congdon N, Jones I, et al. The Lancet Global Health Commission on Global Eye Health: vision beyond 2020. Lancet Glob Health. 2021;9(4):e489-e551.
2. Flaxman SR, Bourne RRA, Resnikoff S, Ackland P, Braithwaite T, Cicinelli MV, et al. Global causes of blindness and distance vision impairment 1990–2020: a systematic review and meta-analysis. The Lancet Global Health. 2017;5(12):e1221-e34.
3. Ting DSJ, Ho CS, Deshmukh R, Said DG, Dua HS. Infectious keratitis: an update on epidemiology, causative microorganisms, risk factors, and antimicrobial resistance. Eye (Lond). 2021;35(4):1084-101.
4. Durand ML, Barshak MB, Chodosh J. Infectious Keratitis in 2021. JAMA. 2021;326(13):1319-20.
5. Ung L, Acharya NR, Agarwal T, Alfonso EC, Bagga B, Bispo PJ, et al. Infectious corneal ulceration: a proposal for neglected tropical disease status. Bull World Health Organ. 2019;97(12):854-6.
6. Brown L, Leck AK, Gichangi M, Burton MJ, Denning DW. The global incidence and diagnosis of fungal keratitis. Lancet Infect Dis. 2021;21(3):e49-e57.
7. Ting DSJ, Ho CS, Cairns J, Elsahn A, Al-Aqaba M, Boswell T, et al. 12-year analysis of incidence, microbiological profiles and in vitro antimicrobial susceptibility of infectious keratitis: the Nottingham Infectious Keratitis Study. Br J Ophthalmol. 2021;105(3):328-33.
8. Collier SA, Gronostaj MP, MacGurn AK, Cope JR, Awsumb KL, Yoder JS, et al. Estimated burden of keratitis--United States, 2010. MMWR Morb Mortal Wkly Rep. 2014;63(45):1027-30.
9. Gopinathan U, Sharma S, Garg P, Rao GN. Review of epidemiological features, microbiological diagnosis and treatment outcome of microbial keratitis: experience of over a decade. Indian J Ophthalmol. 2009;57(4):273-9.

For peer review only

10. Chidambaram JD, Venkatesh Prajna N, Srikanthi P, Lanjewar S, Shah M, Elakkiya S, et al. Epidemiology, risk factors, and clinical outcomes in severe microbial keratitis in South India. Ophthalmic Epidemiol. 2018;25(4):297-305.

11. Ting DSJ, Cairns J, Gopal BP, Ho CS, Krstic L, Elsahn A, et al. Risk Factors, Clinical Outcomes, and Prognostic Factors of Bacterial Keratitis: The Nottingham Infectious Keratitis Study. Front Med (Lausanne). 2021;8:715118.

12. Ting DSJ, Galal M, Kulkarni B, Elalfy MS, Lake D, Hamada S, et al. Clinical Characteristics and Outcomes of Fungal Keratitis in the United Kingdom 2011-2020: A 10-Year Study. J Fungi (Basel). 2021;7(11).

13. Khor WB, Prajna VN, Garg P, Mehta JS, Xie L, Liu Z, et al. The Asia Cornea Society Infectious Keratitis Study: A Prospective Multicenter Study of Infectious Keratitis in Asia. Am J Ophthalmol. 2018;195:161-70.

14. Ting DSJ, McKenna M, Sadiq SN, Martin J, Mudhar HS, Meeney A, et al. Arthrographis kalrae Keratitis Complicated by Endophthalmitis: A Case Report With Literature Review. Eye Contact Lens. 2020;46(6):e59-e65.

15. Ting DSJ, Gopal BP, Deshmukh R, Seitzman GD, Said DG, Dua HS. Diagnostic armamentarium of infectious keratitis: A comprehensive review. Ocul Surf. 2022;23:27-39.

16. Bakken IM, Jackson CJ, Utheim TP, Villani E, Hamrah P, Kheirkhah A, et al. The use of in vivo confocal microscopy in fungal keratitis - Progress and challenges. Ocul Surf. 2022;24:103-18.

17. Ting DSJ, Said DG, Dua HS. Interface Haze After Descemet Stripping Automated Endothelial Keratoplasty. JAMA Ophthalmol. 2019;137(10):1201-2.

18. Khoo P, Cabrera-Aguas MP, Nguyen V, Lahra MM, Watson SL. Microbial keratitis in Sydney, Australia: risk factors, patient outcomes, and seasonal variation. Graefes Arch Clin Exp Ophthalmol. 2020;258(8):1745-55.

19. Ting DSJ, Bignardi G, Koerner R, Irion LD, Johnson E, Morgan SJ, et al. Polymicrobial Keratitis With Cryptococcus curvatus, Candida parapsilosis, and Stenotrophomonas

For peer review only

maltophilia After Penetrating Keratoplasty: A Rare Case Report With Literature Review. Eye Contact Lens. 2019;45(2):e5-e10.

20. Schwalbe N, Wahl B. Artificial intelligence and the future of global health. Lancet. 2020;395(10236):1579-86.
21. LeCun Y, Bengio Y, Hinton G. Deep learning. Nature. 2015;521(7553):436-44.
22. Ting DSW, Cheung CY-L, Lim G, Tan GSW, Quang ND, Gan A, et al. Development and Validation of a Deep Learning System for Diabetic Retinopathy and Related Eye Diseases Using Retinal Images From Multiethnic Populations With Diabetes. JAMA. 2017;318(22):2211-23.
23. De Fauw J, Ledsam JR, Romera-Paredes B, Nikolov S, Tomasev N, Blackwell S, et al. Clinically applicable deep learning for diagnosis and referral in retinal disease. Nat Med. 2018;24(9):1342-50.
24. Seah JCY, Tang CHM, Buchlak QD, Holt XG, Wardman JB, Aimoldin A, et al. Effect of a comprehensive deep-learning model on the accuracy of chest x-ray interpretation by radiologists: a retrospective, multireader multicase study. Lancet Digit Health. 2021;3(8):e496-e506.
25. Soenksen LR, Kassis T, Conover ST, Marti-Fuster B, Birkenfeld JS, Tucker-Schwartz J, et al. Using deep learning for dermatologist-level detection of suspicious pigmented skin lesions from wide-field images. Sci Transl Med. 2021;13(581).
26. Sabanayagam C, Xu D, Ting DSW, Nusinovici S, Banu R, Hamzah H, et al. A deep learning algorithm to detect chronic kidney disease from retinal photographs in community-based populations. Lancet Digit Health. 2020;2(6):e295-e302.
27. Ting DSJ, Ang M, Mehta JS, Ting DSW. Artificial intelligence-assisted telemedicine platform for cataract screening and management: a potential model of care for global eye health. Br J Ophthalmol. 2019;103(11):1537-8.
28. Liu X, Faes L, Kale AU, Wagner SK, Fu DJ, Bruynseels A, et al. A comparison of deep learning performance against health-care professionals in detecting diseases from

For peer review only

medical imaging: a systematic review and meta-analysis. Lancet Digit Health. 2019;1(6):e271-e97.

29. Gulshan V, Peng L, Coram M, Stumpe MC, Wu D, Narayanaswamy A, et al. Development and Validation of a Deep Learning Algorithm for Detection of Diabetic Retinopathy in Retinal Fundus Photographs. JAMA. 2016;316(22):2402-10.

30. Taylor S, Brown JM, Gupta K, Campbell JP, Ostmo S, Chan RVP, et al. Monitoring Disease Progression With a Quantitative Severity Scale for Retinopathy of Prematurity Using Deep Learning. JAMA Ophthalmology. 2019;137(9):1022-8.

31. Li JO, Liu H, Ting DSJ, Jeon S, Chan RVP, Kim JE, et al. Digital technology, tele-medicine and artificial intelligence in ophthalmology: A global perspective. Prog Retin Eye Res. 2021;82:100900.

32. Li W, Yang Y, Zhang K, Long E, He L, Zhang L, et al. Dense anatomical annotation of slit-lamp images improves the performance of deep learning for the diagnosis of ophthalmic disorders. Nat Biomed Eng. 2020;4(8):767-77.

33. Tiwari M, Piech C, Baitemirova M, Prajna NV, Srinivasan M, Lalitha P, et al. Differentiation of Active Corneal Infections from Healed Scars Using Deep Learning. Ophthalmology. 2022;129(2):139-46.

34. Koyama A, Miyazaki D, Nakagawa Y, Ayatsuka Y, Miyake H, Ehara F, et al. Determination of probability of causative pathogen in infectious keratitis using deep learning algorithm of slit-lamp images. Sci Rep. 2021;11(1):22642.

35. Ting DSJ, Foo VH, Yang LWY, Sia JT, Ang M, Lin H, et al. Artificial intelligence for anterior segment diseases: Emerging applications in ophthalmology. Br J Ophthalmol. 2021;105(2):158-68.

36. Li Z, Jiang J, Chen K, Chen Q, Zheng Q, Liu X, et al. Preventing corneal blindness caused by keratitis using artificial intelligence. Nat Commun. 2021;12(1):3738.

37. Hung N, Shih AK, Lin C, Kuo MT, Hwang YS, Wu WC, et al. Using Slit-Lamp Images for Deep Learning-Based Identification of Bacterial and Fungal Keratitis: Model

For peer review only

Development and Validation with Different Convolutional Neural Networks. Diagnostics (Basel). 2021;11(7).

38. Shamseer L, Moher D, Clarke M, Ghersi D, Liberati A, Petticrew M, et al. Preferred reporting items for systematic review and meta-analysis protocols (PRISMA-P) 2015: elaboration and explanation. BMJ. 2015;349:g7647.
39. Salameh J-P, Bossuyt PM, McGrath TA, Thombs BD, Hyde CJ, Macaskill P, et al. Preferred reporting items for systematic review and meta-analysis of diagnostic test accuracy studies (PRISMA-DTA): explanation, elaboration, and checklist. BMJ. 2020;370:m2632.
40. Moons KG, de Groot JA, Bouwmeester W, Vergouwe Y, Mallett S, Altman DG, et al. Critical appraisal and data extraction for systematic reviews of prediction modelling studies: the CHARMS checklist. PLoS Med. 2014;11(10):e1001744.
41. Ouzzani M, Hammady H, Fedorowicz Z, Elmagarmid A. Rayyan-a web and mobile app for systematic reviews. Syst Rev. 2016;5(1):210.
42. Rampat R, Deshmukh R, Chen X, Ting DSW, Said DG, Dua HS, et al. Artificial Intelligence in Cornea, Refractive Surgery, and Cataract: Basic Principles, Clinical Applications, and Future Directions. Asia Pac J Ophthalmol (Phila). 2021;10(3):268-81.
43. Whiting PF, Rutjes AW, Westwood ME, Mallett S, Deeks JJ, Reitsma JB, et al. QUADAS-2: a revised tool for the quality assessment of diagnostic accuracy studies. Ann Intern Med. 2011;155(8):529-36.
44. Sounderajah V, Ashrafian H, Rose S, Shah NH, Ghassemi M, Golub R, et al. A quality assessment tool for artificial intelligence-centered diagnostic test accuracy studies: QUADAS-AI. Nat Med. 2021;27(10):1663-5.
45. Campbell JM, Klugar M, Ding S, Carmody DP, Hakonsen SJ, Jadotte YT, et al. Diagnostic test accuracy: methods for systematic review and meta-analysis. JBI Evidence Implementation. 2015;13(3).

For peer review only

46. Deeks JJ, Macaskill P, Irwig L. The performance of tests of publication bias and other sample size effects in systematic reviews of diagnostic test accuracy was assessed. J Clin Epidemiol. 2005;58(9):882-93.

47. van Enst WA, Ochodo E, Scholten RJ, Hooft L, Leeflang MM. Investigation of publication bias in meta-analyses of diagnostic test accuracy: a meta-epidemiological study. BMC Med Res Methodol. 2014;14:70.

For peer review only

PRISMA-P (Preferred Reporting Items for Systematic review and Meta-Analysis Protocols) 2015 checklist: recommended items to address in a systematic review protocol*

Section and topic	Item No	Checklist item	Page
**ADMINISTRATIVE INFORMATION**			
Title:			
Identification	1a	Identify the report as a protocol of a systematic review	1
Update	1b	If the protocol is for an update of a previous systematic review, identify as such	N/A
Registration	2	If registered, provide the name of the registry (such as PROSPERO) and registration number	4
Authors:			
Contact	3a	Provide name, institutional affiliation, e-mail address of all protocol authors; provide physical mailing address of corresponding author	2
Contributions	3b	Describe contributions of protocol authors and identify the guarantor of the review	14
Amendments	4	If the protocol represents an amendment of a previously completed or published protocol, identify as such and list changes; otherwise, state plan for documenting important protocol amendments	N/A
Support:			
Sources	5a	Indicate sources of financial or other support for the review	14
Sponsor	5b	Provide name for the review funder and/or sponsor	14
Role of sponsor or funder	5c	Describe roles of funder(s), sponsor(s), and/or institution(s), if any, in developing the protocol	14
**INTRODUCTION**			
Rationale	6	Describe the rationale for the review in the context of what is already known	5-6
Objectives	7	Provide an explicit statement of the question(s) the review will address with reference to participants, interventions, comparators, and outcomes (PICO)	7
**METHODS**			
Eligibility criteria	8	Specify the study characteristics (such as PICO, study design, setting, time frame) and report characteristics (such as years considered, language, publication status) to be used as criteria for eligibility for the review	7-8
Information sources	9	Describe all intended information sources (such as electronic databases, contact with study authors, trial registers or other grey literature sources) with planned dates of coverage	8
Search strategy	10	Present draft of search strategy to be used for at least one electronic database, including planned limits, such that it could be repeated	8

For peer review only

Study records:			
Data management	11a	Describe the mechanism(s) that will be used to manage records and data throughout the review	9
Selection process	11b	State the process that will be used for selecting studies (such as two independent reviewers) through each phase of the review (that is, screening, eligibility and inclusion in meta-analysis)	8-9
Data collection process	11c	Describe planned method of extracting data from reports (such as piloting forms, done independently, in duplicate), any processes for obtaining and confirming data from investigators	10
Data items	12	List and define all variables for which data will be sought (such as PICO items, funding sources), any pre-planned data assumptions and simplifications	10
Outcomes and prioritization	13	List and define all outcomes for which data will be sought, including prioritization of main and additional outcomes, with rationale	10-11
Risk of bias in individual studies	14	Describe anticipated methods for assessing risk of bias of individual studies, including whether this will be done at the outcome or study level, or both; state how this information will be used in data synthesis	11
Data synthesis	15a	Describe criteria under which study data will be quantitatively synthesised	12
	15b	If data are appropriate for quantitative synthesis, describe planned summary measures, methods of handling data and methods of combining data from studies, including any planned exploration of consistency (such as I^2, Kendall's τ)	12
	15c	Describe any proposed additional analyses (such as sensitivity or subgroup analyses, meta-regression)	12
	15d	If quantitative synthesis is not appropriate, describe the type of summary planned	12-13
Meta-bias(es)	16	Specify any planned assessment of meta-bias(es) (such as publication bias across studies, selective reporting within studies)	13
Confidence in cumulative evidence	17	Describe how the strength of the body of evidence will be assessed (such as GRADE)	13

*** It is strongly recommended that this checklist be read in conjunction with the PRISMA-P Explanation and Elaboration (cite when available) for important clarification on the items. Amendments to a review protocol should be tracked and dated. The copyright for PRISMA-P (including checklist) is held by the PRISMA-P Group and is distributed under a Creative Commons Attribution Licence 4.0.**

From: Shamseer L, Moher D, Clarke M, Ghersi D, Liberati A, Petticrew M, Shekelle P, Stewart L, PRISMA-P Group. Preferred reporting items for systematic review and meta-analysis protocols (PRISMA-P) 2015: elaboration and explanation. BMJ. 2015 Jan 2;349(jan02 1):g7647.

For peer review only
